# Origin of the Optimization of Photocatalytic Activities for Titanium Oxide Film Modified by an Oxidized Copper Layer

**DOI:** 10.3390/ma18132993

**Published:** 2025-06-24

**Authors:** Jian-An Chen, Shu-Min Tsai, Yi-You Hong, Pin-Jyun Shih, Day-Shan Liu

**Affiliations:** Institute of Electro-Optical and Materials Science, National Formosa University, Huwei 63201, Taiwan

**Keywords:** photocatalytic activity, TiO_x_/Cu structure, antibacterial activity, *n/p* nanocomposite heterojunction, *p*-type cuprous oxide

## Abstract

In this study, the surface photocatalytic activity of an anatase–titanium oxide (TiO_x_) film was modified by a thin copper (Cu) layer with the subsequential oxidation annealing process. Through this simple annealing process, the photocatalytic activity of the TiO_x_/Cu structure to decompose the methylene blue solution and inhibit the growth of *Escherichia coli*. could be optimized. With the help of a study on the conductive type required for the oxidation of a single Cu layer, an *n/p* nanocomposite heterojunction was realized, as this contact system anneals at temperatures of 350 °C and 450 °C. An extra electrical field at the contact interfaces that was be beneficial for separating the photo-generated electron–hole pairs (EHPs) under UV light irradiation was built. The built-in electrical field led to an increase in the structural photocatalytic activity. Moreover, as the *p*-type cuprous oxide (*p*-Cu_2_O) structure oxidized by the annealed Cu layer could provide a high conduction band that is offset when in contact with the TiO_x_ film, the photogenerated EHPs on the TiO_x_ surface could be separated more effectively. Accordingly, the 350 °C-annealed sample, abundant in the nanocomposite TiO_x_/Cu_2_O heterojunction which could significantly retard the recombination of photo-generated carriers, corresponded to an increase of about 38% in the photocatalytic activity as compared with the single TiO_x_ film.

## 1. Introduction

Metal oxide films with controllable conduction and broadband transparency are quite appropriate for using with optical and electrical coatings. Among those coatings, titanium oxide (TiO_x_) possesses a high refractive index with wide energy bandgap, making it an ideal candidate to be applied in the laminated optical structure, both at visible wavelengths and, in electronics, as a transparent dielectric layer [1,2,3]. Moreover, TiO_x_ has low toxicity and bio-inertness as well as strong redox ability when illuminated by ultraviolet (UV) radiation, leading it to become a valuable surface modification layer in the environmental purification and biomedical engineering applications [4,5,6,7]. Redox complexes with strong oxidative and reduction potential, such as hydroxyl radical (·OH), superoxide radical (·O_2_^−^), and hydrogen peroxide (H_2_O_2_), form on the UV-light-activated TiO_x_ surface as a consequence of the photo-generated electron–hole pairs (EHPs), reacting with the environmental vapor and oxygen to provide excellent photocatalysis for mineralizing pollutants and inhibiting bacterial growth. Thus far, researchers have sought to achieve a TiO_x_ film that possesses the anatase crystalline structure with a maximized specific surface area through adequate deposition and/or post-annealing processes that behave as a nanotextured surface [8,9,10]. In addition to engineering the TiO_x_ film’s crystallinity and surface morphology, researchers have also tried to optimize the photocatalytic activity of the TiO_x_ film in visible-light wavelengths by doping specific impurities [11,12,13]. Another effective method for enhancing the photocatalytic activity of a TiO_x_ film is building a heterojunction structure for inhibiting the recombination of the photo-generated EHPs on the TiO_x_ film’s surface. To achieve this, noble metal that possesses a high Fermi energy level (e.g., Au, Ag, and Pt), serving as a sink for the photo-generated electrons, is applied to create hetero-contact with the TiO_x_ film [14,15,16]. In our previous study, a thin Ag layer was deposited onto the TiO_x_ film to modify the structural photocatalytic activity. It was found that the TiO_x_ film surface decorated by the Ag nanoparticle achieved by an annealing process under ambient nitrogen possessed the optimal photocatalytic activity [17]. In addition to engineering the hetero-contact by using the noble metal and TiO_x_, *p*-type semiconductors oxidized from the d-block transition metals are another promising material for enhancing structural photocatalytic activity that occurs while in contact with a TiO_x_ film [18,19,20,21]. This heterojunction structure realizes the *p/n* junction structure, which can build an internal electric field for facilitating the separation of the photo-generated EHPs. Among these structures, *p*-type copper oxide (Cu_x_O) is the preferred candidate because the element Cu is one of the most abundant transition metals and is more cost-effective than the noble metals. To obtain the Cu_x_O film, the thermal oxidation process on the metallic Cu layer is the simplest and most cost-effective method. Given that, on the thermal oxidized Cu species, *p*-type semiconducting cupric oxide (CuO) and cuprous oxide (Cu_2_O) are the main crystalline phases and given the different physical properties in these two *p*-type semiconductors, the content of these two phases (Cu_2_O/CuO) in the TiO_x_/Cu_x_O heterojunction contact system becomes a key issue for influencing the resulting photocatalytic activity. For instance, Kim et al. synthesized a mesoporous copper oxide–titanium oxide nanohybrid structure using a solid-state reaction process. The authors found that post-heated temperatures (200–500 °C) are crucial for the phase transformation of the rutile and anatase TiO_2_ structures as well as the oxidation phases of the Cu_2_O and CuO structures. The 200 °C-calcined CuO/Cu_2_O-TiO_2_ nanohybrid structure irradiated by visible light showed optimal photocatalytic activity for decomposing the methyl orange [22]. Yurddaskal et al. prepared Cu_2_O and CuO structures from the electroplated Cu coatings on the titanium substrate using thermal annealing in ambient air from 200 °C to 600 °C. They found that the CuO/Cu_2_O structures achieved from the 500 °C-annealed process demonstrated the highest photodegradation efficiency for MB solution. The reason for the optimal photocatalytic activity was the enriched surface structure of the CuO phase, which is beneficial for the interfacial charge when transferring and inhibiting the recombination of the photo-generated EHPs [23]. More recently, Sarac et al. produced Cu_x_O nanowires from thermal oxidized copper foil at temperatures ranging from 400 °C to 600 °C. The authors subsequently coated the foil with TiO_2_ film via the hydrolysis process using a titanium isopropoxide solution to prepare the Cu_x_O/TiO_x_ structure so as to improve the charge separation efficiency under UV-A light irradiation. They found that the photocatalytic activity was highly dependent on the CuO nanowire density, nanowire aspect ratio, and the TiO_2_ layered thickness. This Cu_x_O nanowire/TiO_2_ structure with an optimal photocatalytic performance was obtainable from the 450 °C-annealed Cu_x_O nanostructures immersed in 40 mM solution [24].

According to the above-mentioned reports, the photocatalytic performance of the TiO_x_/Cu_x_O system is deeply correlated to its post-annealing temperatures, which are crucial to the appearance of the *p*-type CuO and Cu_2_O phases. Here, with the aim to simplify the fabrication and develop a TiO_x_/Cu heterojunction structure without the effect on the substate’s scale or shape, we engineered a conventional thin-film deposition method with a post-annealing treatment to achieve the TiO_x_/Cu_x_O contact system. In order to accomplish this objective, the TiO_x_ film with anatase phase that had been affected by the CuO and Cu_2_O crystallization by a simple post-oxidation treatment on the evaporated thin Cu layer was carried out and investigated. The corresponding photocatalytic and antibacterial activities were determined as the as-deposited and annealed samples were used to decompose the methylene blue (MB) solution and sterilize *E. coli* while being activated by irradiation using a UV lamp. The chemical bond configurations at the interface of the TiO_x_/Cu contact system, as well as the crystalline and phase transformation of the single Cu layer oxidized by the identical annealing process, were comprehensively studied to elucidate the evolutions of the resulting photocatalytic activities. Based on these investigations, the mechanism responsible for the TiO_x_/Cu structure through a simple oxidation process that exhibited the optimal photocatalytic activity was conducted. Such a TiO_x_/Cu structure, prepared purely by the thin-film deposition technology, has the advantage of thickness uniformity and material consistency over a large area, leading it to become less susceptible to the substrate/device size and shape than similar structures that are reported in the referenced papers.

## 2. Material Preparation and Experimental Procedure

Amorphous titanium oxide (TiO_x_) films with a thickness of 200 nm were deposited onto n-type silicon (100) and quartz substrates, using the plasma enhanced chemical vapor deposition (PECVD) system under an ambient temperature of 200 °C. A titanium tetraisopropoxide [Ti(OC_3_H_7_)_4_, TTIP] liquid source [97%, Sigma-Aldrich, Saunt Louis, MO, USA] was utilized as the titanium precursor through a heated bubbling cylinder at 70 °C and carried by nitrogen gas. A vaporized TTIP precursor was mixed with oxygen gas in a cylindrical stainless-steel barrel. All the gas pipelines were heated to 100 °C to prevent the condensation of the precursor and gas mixture. The deposition pressure, rf power, and TTIP/O_2_ gas mixture were controlled at 40 Pa, 100 W, and 120/20 sccm, respectively. Detailed deposition parameters and system setup are described elsewhere [25]. The deposited TiO_x_ film was then annealed at 500 °C for 30 min under ambient oxygen to result in the anatase crystallization [25]. A Cu layer with a thickness of about 10 nm was then evaporated onto the anatase–TiO_x_ film surface, and sequentially annealed at 250 °C, 350 °C, and 450 °C under ambient oxygen for 1 min. To give an insight into the evolutions of the electrical and crystalline properties of the Cu layer itself, another set of the single Cu layer with a thickness of about 200 nm was directly deposited onto the silicon and glass substrates and then processed by the same rapidly thermal annealing (RTA) treatment at a temperature ranging from 250 °C to 450 °C for 1 min under ambient oxygen.

The film thickness of the TiO_x_ film, the Cu layer, and the TiO_x_/Cu heterojunction structure was measured using a surface profile system (Dektak 6M, Veeco, Plainview, NY, USA) and confirmed by cross-section field emission scanning electron microscope (FE-SEM, JSM-6700F, JEOL, Akishima, Tokyo) images. Surface roughness and morphologies were examined using atomic force microscopy (AFM, DI-3100, Veeco, Plainview, NY, USA) and plane view FE-SEM observations. The optical transmittance of these samples was conducted with a UV–Vis–NIR spectrophotometer (UVD 3500, Labomed, Inc., Los Angeles, CA, USA). The crystallinities of the TiO_x_/Cu structures and of the single Cu layer oxidized under the same annealing treatments were analyzed using a grazing incident X-ray diffractometer (GIXRD) at 30 kV and 30 mA using Cu *K_α_*_1_ radiation (D-500, Siemens, Munich, Germany). An X-ray photoelectron spectroscope (XPS) with monochromatic Al *Kα* radiation (PHI Quantera SXM^TM^, ULVAC-PHI, Chigasaki, Japan) was employed to examine the chemical bond configurations distributed over the interface of the as-deposited and annealed TiO_x_/Cu contact systems. Ar^+^ sputtering was used to etch the TiO_x_/Cu structure to the depth near the contact interface during the XPS measurements. The XPS signal at the binding energy of 284.6 eV in the C 1*s* core level, which occurred due to the surface hydrocarbon contaminations, served as the reference to predetermine the adequacy of the charge neutralization. The binding energy of the Ti 2*p* core level examined from the single TiO_x_ film was used as an internal reference signal to calibrate the XPS spectra examined from these TiO_x_/Cu structures after etching by the Ar^+^ sputtering [26,27]. The electrical properties of the single Cu layer treated by the same RTA process were measured using van der Pauw Hall measurements (Ecopia HMS-5000, Ecopia, Anyang, Republic of Korea) at room temperature. The resulting photocatalytic activities of the as-deposited and annealed TiO_x_/Cu structures, as well as the single TiO_x_ and Cu films, were evaluated from these measurements in order to decolorize an aqueous MB solution (20 mgL^−1^ with a pH value approximately of 7.0 ± 0.1) at ambient temperature under UV lamp irradiation. The associated antibacterial activities were assessed with a plate-counting method while the samples were used against *E. coli*. Microbiological tests were carried out as the samples were immersed into a nutrient broth, and the initial concentration of the *E. coli* bacteria was adjusted to 1.0 × 10^6^ colony-forming unit (CFU)/mL by dilution. These specimens were then sterilized under UV lamp irradiation for 1 h. Subsequently, 0.1 mL of each dilution was taken and spread on the nutrient agar and then incubated at 37 °C for 24 h. The number of bacterial colonies grown on the plates was counted (in CFU) and photographed. The UV lamp emitted the dominant wavelength of 365 nm with a power density that was controlled at 5 mW/cm^2^. The photo-excited currents—generated as the UV lamp irradiated on to the surface of the single TiO_x_ film and TiO_x_/Cu heterojunction structures with the pattern of the interdigital transducer (IDT) electrodes while operated at a controlled bias of 10 V—were also measured using a semiconductor parameter analyzer (HP4156C, Keysight Technology, Santa Clara, CA, USA). Based on the specific analysis of the crystallinity of the single Cu layer annealed by the same oxidation treatment, the mechanism responsible for the changes of the photocatalytic activities for these TiO_x_/Cu contact systems treated through the simple annealing process can be elucidated more clearly.

## 3. Results and Discussions

**Figure 1:**
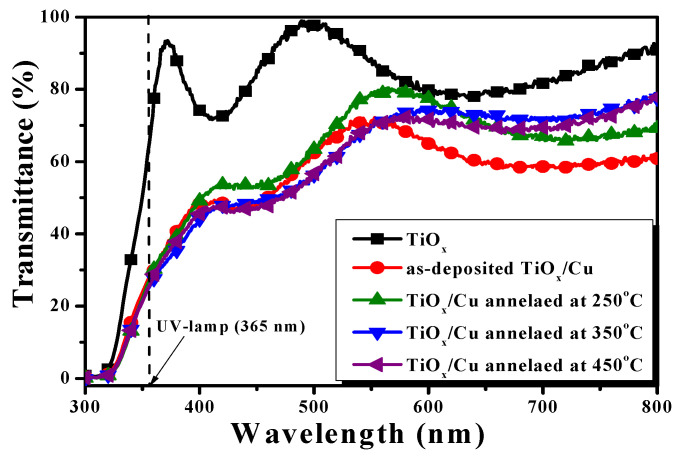
Optical transmittance of a 10 nm thick Cu layer deposited onto the TiO_x_ film and sequentially treated by an RTA process at temperatures of 250 °C, 350 °C, and 450 °C for 1 min under ambient oxygen, as well as the single TiO_x_ film.

The optical transmittance of the Cu layer deposited onto the TiO_x_ film and sequentially treated by an RTA process at temperatures of 250 °C, 350 °C, and 450 °C for 1 min under ambient oxygen, as well as the single TiO_x_ film, are illustrated in Figure 1. The TiO_x_ film showed optical transparency, with an average transmittance of about 84% around the visible wavelength (from 400 nm to 700 nm). Beyond the visible wavelength, a drastic decrease in the optical transmittance at the onset of the ultraviolet wavelength (~370 nm) due to the film’s absorption originating from the energy bandgap can be seen. As a 10 nm thick Cu layer deposited onto the TiO_x_ film, an apparent reduction in the optical transmittance and redshift of the absorption edge was observed. The opaque nature of the Cu metal led to the average transmittance around the visible wavelength decreasing to about 60%. For the TiO_x_/Cu structure treated by the RTA process, the average transmittance around the visible wavelengths was somewhat higher than that of the as-deposited TiO_x_/Cu structure, while the absorption edge at the UV wavelength of these annealed samples was almost the same as the as-deposited one. This result reveals that the thin metallic Cu and the oxidized Cu layer covered on the TiO_x_ film both showed a similar obstruct feature as at the visible light, it also occurred in the marked redshift of the absorption edge when compared with that of the single TiO_x_ film.

**Figure 2:**
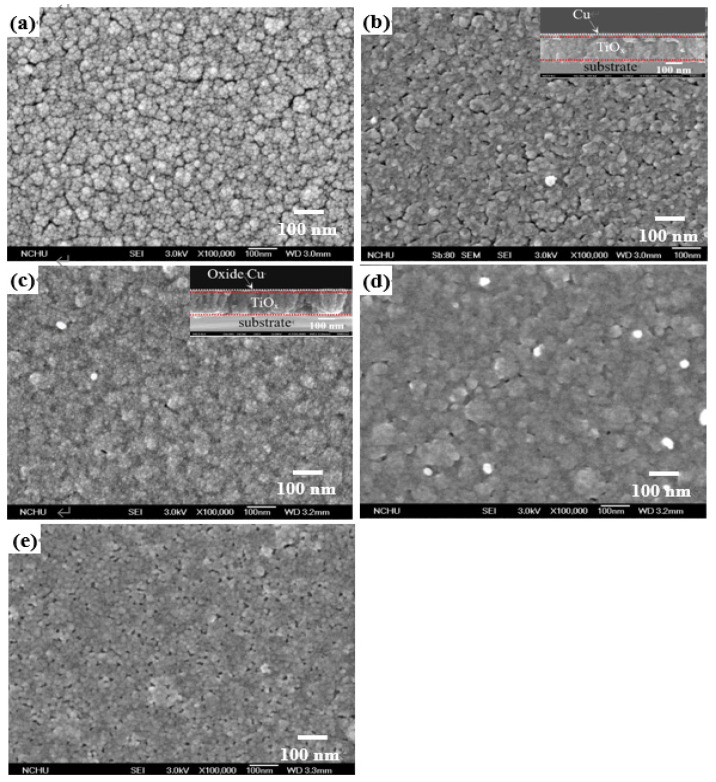
Surface morphologies of the (**a**) TiO_x_ film and the (**b**) as-deposited TiO_x_/Cu structure, and the TiO_x_/Cu structures annealed at temperatures of (**c**) 250 °C, (**d**) 350 °C, and (**e**) 450 °C (the cross-sectional images of the as-deposited and 250 °C-annealed samples are also shown in the inset figures).

Figure 2a–e respectively show the surface morphologies of the TiO_x_ film and the TiO_x_/Cu structures before and after processing the RTA treatment at temperatures of 250 °C, 350 °C, and 450 °C (the cross-sectional images of the as-deposited and 250 °C-annealed TiO_x_/Cu structure are also shown in the inset figures). The surface of the anatase–TiO_x_ film (Figure 2a) was uneven, with irregular grains and visible boundaries. Those fine grains distributed over the TiO_x_ surface became invisible as the film was covered by a 10 nm thick Cu layer (Figure 2b). The grain boundaries distributed over the surface were then hardly evident, resulting in an ambiguous surface morphology as the TiO_x_/Cu structure annealed at 250 °C (Figure 2c). The surface morphology of the 350 °C-annealed TiO_x_/Cu structure (Figure 2d) became more ambiguous with the appearances of voids and white protrusions. When the annealed temperature reached 450 °C, as shown in Figure 2e, the voids that might be relevant to the outdiffusion of the oxidized Cu layer were comprehensively distributed over the structural surface. Moreover, although the layered structure could be roughly defined from the inset figures, the change in the microstructures at the TiO_x_/Cu interface affected by the annealing process was hardly observed.

**Figure 3:**
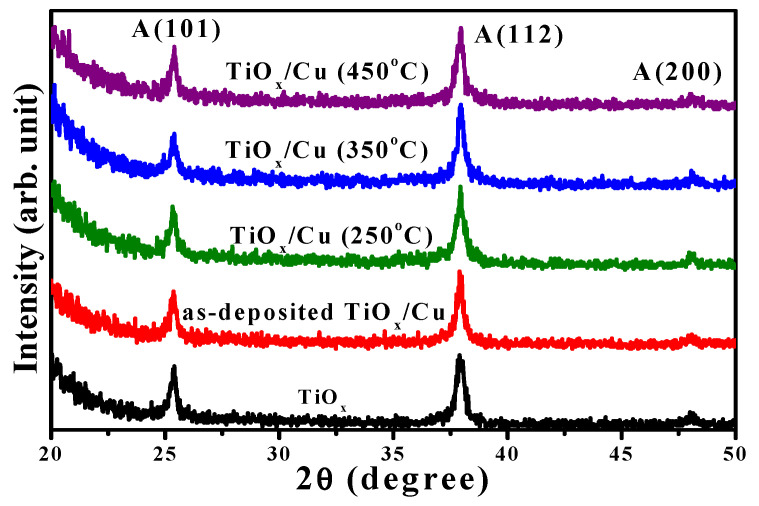
X-ray diffraction spectra of the single TiO_x_ film, the as-deposited TiO_x_/Cu structure, and the TiO_x_/Cu structures annealed at temperatures of 250 °C, 350 °C, and 450 °C (the diffraction peaks for the anatase phase are marked as “A,” with the crystal planes (hkl)).

The crystallinity of the as-deposited and annealed TiO_x_/Cu structures, as well as the single TiO_x_ film conducted from the XRD measurements, are presented in Figure 3. The TiO_x_ film formed the polycrystal structure with only the anatase phase of the two apparent crystal planes, (hkl), of (101) and (112) and a weak plane of (200) (the diffraction peaks for the anatase phase are taken from the JCPDS No.021-1272 and are marked as “A,” with the crystal plane (hkl) also given). As thin Cu layers coated onto the TiO_x_ film, the diffraction peaks and their positions associated with different crystal planes of the anatase structure in the XRD spectrum were basically identical to the single TiO_x_ film. For the TiO_x_/Cu structures annealed at temperatures of 250 °C, 350 °C, and 450 °C, the resulting respective XRD patterns still exhibited the anatase phase with dominant crystal planes of (101) and (112). The intensity and position of these two crystal planes were almost identical to that of the single TiO_x_ film. This result reveals that neither the metallic Cu deposition nor the following oxidation process on the TiO_x_/Cu structure affected the crystallinity of the under-layered TiO_x_ film. Moreover, no clear evidence of the Cu atoms being doped into the TiO_x_ matrix emerged, as the peak positions of the two anatase planes that appeared in the XRD spectra were basically duplicates of the single TiO_x_ film. In addition to the investigations on the crystallinity of the under-layered TiO_x_ film, no featured peaks related to the upper-layered Cu could be identified from these XRD spectra. This might be because the signal related to the metallic Cu and/or its oxidized states was too weak to be observed by the XRD measurements. Accordingly, further observation on the changes of the metallic Cu layer oxidized by the annealing process was essential.

**Figure 4:**
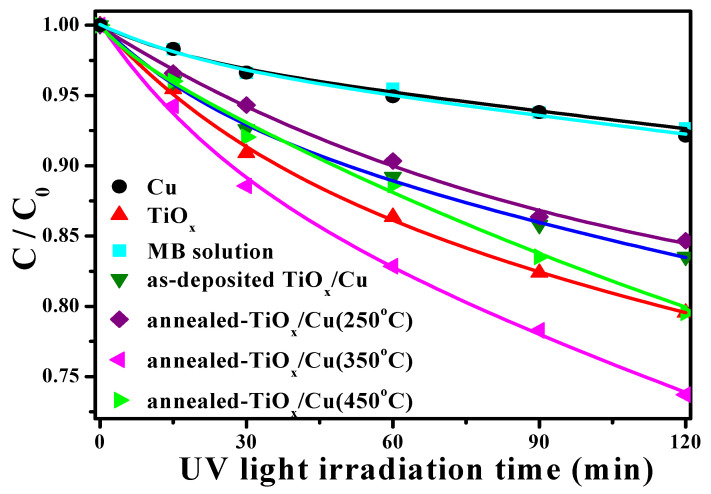
Concentration evolution of the MB solution as a function of UV light irradiation time catalyzed using the single Cu and TiO_x_ films, and the as-deposited and annealed TiO_x_/Cu structures (the change on the concentration of the MB solution directly irradiated by the UV light is also given as a comparison).

Figure 4 represents the degradation rate associated with the concentration evolution on the MB solution as a function of the UV light irradiation time catalyzed using the single Cu and TiO_x_ films as well as the as-deposited and annealed TiO_x_/Cu structures (the change in the concentration of the MB solution directly irradiated by the UV light is also given as a comparison). According to the limited degradation rate in the MB concentration, as the irradiation time increased, both the MB solution itself and the solution catalyzed by the single 200 nm thick Cu film were only slightly reactive to the UV light illumination. In contrast, the MB solution decomposed by the UV light irradiation, together with the TiO_x_ film catalyzation, was significantly enhanced, as could be seen in the marked reduction in the concentration rate as the irradiation time increased. As a thin Cu layer covered the TiO_x_ film, the resulting decomposition efficiency catalyzed by this TiO_x_/Cu structure became apparently inferior to the TiO_x_ film, even when this structure annealed at a temperature of 250 °C. In contrast to the as-deposited and 250 °C-annealed TiO_x_/Cu structures, the UV light-induced decomposition to the MB solution incorporating the 350 °C-annealed TiO_x_/Cu structure was greatly enhanced, which demonstrates that the degradation rate was clearly faster than that of the MB solution photo-catalyzed only by the TiO_x_ film. The resulting degradation rate of the MB solution catalyzed using the TiO_x_/Cu structure after being annealed at an elevated temperature of 450 °C was again not as good as the degradation rate using the single TiO_x_ film. The rate constant, *k*, which is derived by fitting the concentration evolution of the MB solution under UV light irradiation, complied with the first-order rate equation, which is addressed as the following equation that was employed to quantify the decomposition ability of the MB solution [28]:

(1) $$\ln\left(\frac{C}{C_{0}}\right)=kt$$

where *C* and *C_o_* are the concentrations of the MB solution under UV light irradiation times of *t* = 0 and *t*, respectively. The rate constant values analyzed from the curves in Figure 4 are summarized in Table 1. The decomposition of the MB solution catalyzed by the Cu film under UV light irradiation corresponded to a rate constant (~0.0038 min^−1^) similar to the value derived from the MB solution directly decomposed by UV light illumination (~0.0036 min^−1^). This decomposition increased markedly to about 0.0120 min^−1^ as the solution was photo-decomposed by incorporating the single TiO_x_ film. The value decreased to about 0.0091 min^−1^ as the solution catalyzed when using the TiO_x_ film covered by a 10 nm thick Cu layer, and a low value of about 0.0087 min^−1^ was derived from the degradation rate of the MB solution using the 250 °C-annealed TiO_x_/Cu structure. When the TiO_x_/Cu structure was annealed at a temperature of 350 °C, the specimen used to photo-decompose the MB solution corresponded to the highest rate constant of about 0.0165 min^−1^. It then decreased to about 0.0118 min^−1^, as derived from the MB solution photo-decomposed by using the TiO_x_/Cu structure annealed at an elevated temperature of 450 °C. This result indicates that the UV-activated degradation rate of the MB solution could be enhanced by more than three times as it was photo-catalyzed using the single TiO_x_ film. The degradation efficiency of the MB solution could be further improved by about 40% with the assistance of an oxidized Cu layer annealed at 350 °C, while other annealed treatments on the Cu layer showed little support or even obstructed the TiO_x_ film’s ability to photo-decompose the MB solution.

**Figure 5:**
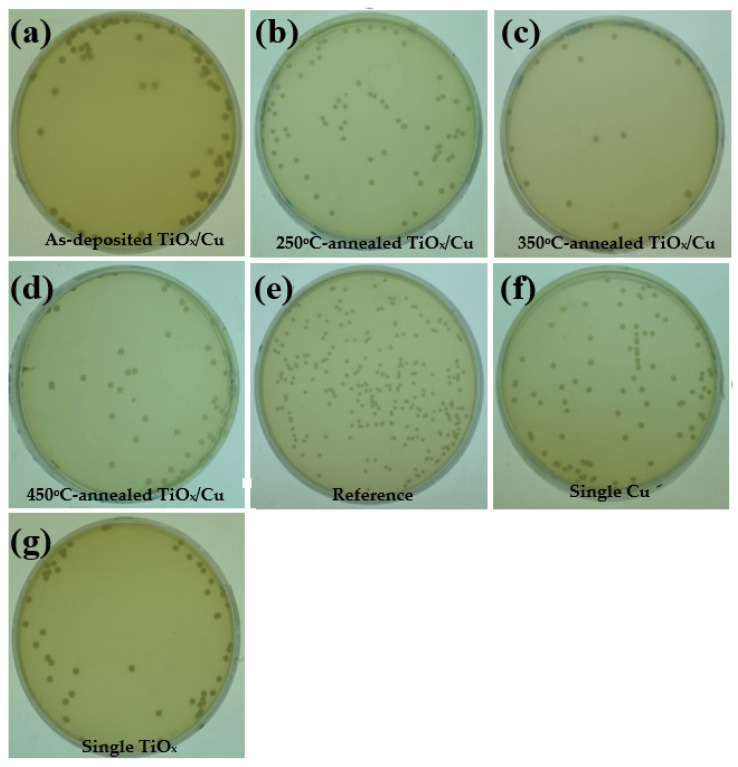
Photographs of the *E. coli* bacterial colonies on the nutrient agar after a 24 h incubation period, sterilized using the (**a**) as-deposited, (**b**) 250 °C-, (**c**) 350 °C-, and (**d**) 450 °C-annealed TiO_x_/Cu UV light-activated structures, respectively (images of the *E. coli* bacterial colonies directly illuminated by the UV light (**e**) (reference) as well as the *E. coli* bacterial colonies that were sterilized and incorporated with the single Cu (**f**) and TiO_x_ (**g**) films are also given as a comparison).

Figure 5a–d respectively show the photographs of the *E. coli* bacterial colonies on the nutrient agar after a 24 h incubation period, sterilized using the UV light-activated as-deposited and annealed TiO_x_/Cu structures. The photographs of the plate for the *E. coli* bacterial colonies that were directly illuminated by the UV light (Figure 5e, the *E. coli* bacterial colonies sterilized using the single Cu (Figure 5f) and TiO_x_ (Figure 5g) films are also given as a comparison). Although the UV-activated Cu film was inactive while decomposing the MB solution, it showed the functional ability to inhibit the growth of the *E. coli* bacteria. The number of bacterial colonies counted from the plate (Figure 5f) decreased to about (87 ± 5.2) × 10^3^ CFU/mL, whereas the *E. coli* bacteria directly illuminated by the UV light irradiation was about (138 ± 5.5) × 10^3^ CFU/mL (Figure 5e). The growth of the *E. coli* bacteria could be further controlled to about (50 ± 4.9) × 10^3^ CFU/mL while the Cu film was substituted by the TiO_x_ film (Figure 5g). These results indicate that the UV-activated Cu and TiO_x_ films are both beneficial for suppressing the growth of *E. coli* bacteria. When a thin copper layer was coated onto the TiO_x_ film, the resulting bacterial colonies appearing on the plate, as shown in Figure 5a, were about (64 ± 4.9) × 10^3^ CFU/mL. Although this value is somewhat higher than that of the bacterial colonies using the single TiO_x_ film, the suppressed efficiency in the growth of the *E. coli* bacteria is still apparent when compared with the sterilization using only the single Cu film. Thus, it can be concluded that the under-layered TiO_x_ film in the TiO_x_/Cu structure also played an important part in inhibiting the growth of the *E. coli* bacteria. As the TiO_x_/Cu structure annealed at a temperature of 250 °C, the growth of the bacterial colonies that appear in Figure 5b was slightly increased to about (79 ± 5.0) × 10^3^ CFU/mL). By contrast, the growth of the bacterial colonies could be minimized to about (12 ± 2.1) × 10^3^ CFU/mL, as the *E. coli* bacteria were sterilized using the 350 °C-annealed TiO_x_/Cu structure (Figure 5c). An increase in the number of bacterial colonies (~(45 ± 3.5) × 10^3^ CFU/mL) was observed from the plate sterilized using the 450 °C-annealed TiO_x_/Cu structure (Figure 5d). The reduction percentage, *R*, which quantitatively represents the antibacterial activity of the specimen under UV lamp irradiation, is determined by the evolution of the bacterial colonies on the plate using the following equation [29]:

(2) $$R\;\left(\%\right)=\left[\left(A-B\right)/A\right]\times 100\%$$

where *A* is the number of the bacterial colonies counted from the *E. coli* directly sterilized by the UV lamp irradiation and *B* is the number of the bacterial colonies counted from the plate treated by the specimen incorporation. The higher the reduction percentage, the better the antibacterial activity. Table 1 summarizes the reduction percentages of the plates shown in Figure 5. According to this table, the antibacterial activity of the single TiO_x_ film (~64%) was almost twice as high as the single Cu film (~37%). The degree for the antibacterial activity of the TiO_x_ film decreased somewhat to about 54% as a semi-opaque Cu layer covered the TiO_x_ film. The structural activity to sterilize the *E. coli* bacterial was continuously deteriorated (~43%) as it was annealed at a temperature of 250 °C. In contrast to the 250 °C-annealed sample, a remarkable increase in antibacterial activity (~91%) was obtained from the TiO_x_/Cu structure annealed at a temperature of 350 °C. The antibacterial activity was again decreased to about 68% while the annealed temperature on the TiO_x_/Cu structure reached 450 °C. According to the values listed in Table 1, although the degrees of the rate constant and reduction percentage using the single Cu film were both inferior to those when using the single TiO_x_ film, the photocatalytic and antibacterial activities for the Cu coated onto the TiO_x_ film could still be optimized as this structure annealed at a temperature of 350 °C.

**Figure 6:**
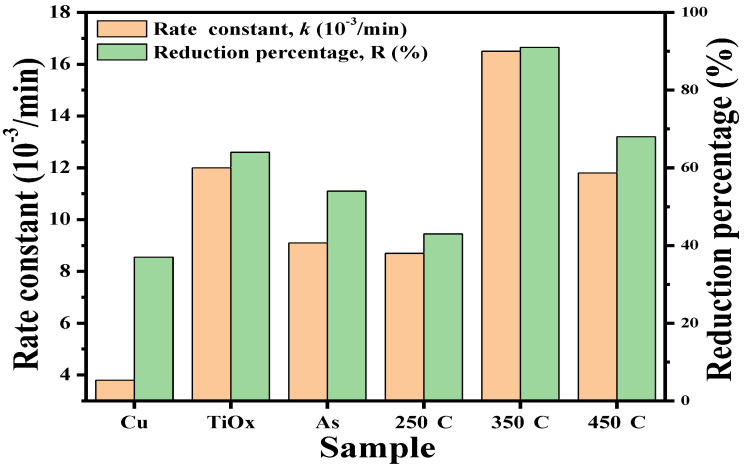
Rate constant and reduction percentage of the as-deposited and annealed TiO_x_/Cu contact systems as well as the single Cu and TiO_x_ films.

Figure 6 depicts the rate constant and reduction percentage of the as-deposited and annealed TiO_x_/Cu contact systems as well as the single Cu and TiO_x_ films listed in Table 1. The inactive behavior of the single Cu layer and the good photocatalytic activity of the anatase–TiO_x_ film under UV light irradiation resulted from the formation of the surface hydroxyl radicals that could be clearly seen from the significant difference in the values of their rate constants. However, though the Cu layer was inactive to the UV light irradiation, the metallic Cu showed an ability to inhibit the growth of the *E. coli* as a consequence of the bacterial cell being broken by the Cu ion and this ability corresponded to a reduction percentage of 37%. When a thin Cu layer is coated onto the TiO_x_ film, this structure becomes activable by the UV light irradiation, as observed in the comparison of its rate constant to that of the single Cu layer. However, the shielding effect of the UV light by the surface Cu layer results in an inferior rate constant than the single TiO_x_ layer. In contrast, the photocatalytic activity induced by the under-layered TiO_x_ film was found to be beneficial for enhancing the structural antibacterial ability to a value of 54% as compared with that of the single Cu layer. It can also be seen that there was only little change in the rate constant from the 250 °C-annealed TiO_x_/Cu structure, while an obvious decrease in the reduction percentage was derived. This reveals that the UV-activated property of this structure was nearly changed, but that the amount of the Cu ions required to break the bacteria had been significantly reduced as a consequence of the oxidation treatment. Both the rate constant and reduction percentage were optimized as the TiO_x_/Cu structure was annealed at 350 °C, and these values decreased again when the structure was annealed at a temperature of 450 °C. This suggests that a different mechanism dominated the increase in the photocatalytic activities of these annealed TiO_x_/Cu contact systems. As there was neither a change in the crystallinity nor evidence of a new compound or dopant formation in the TiO_x_ film, the ability of the annealing process on the oxidation of the upper-layered Cu to affect the TiO_x_/Cu contact interface might be crucial for determining the structural activities required to decompose the MB solution as well as sterilize the *E. coli* bacterial.

**Figure 7:**
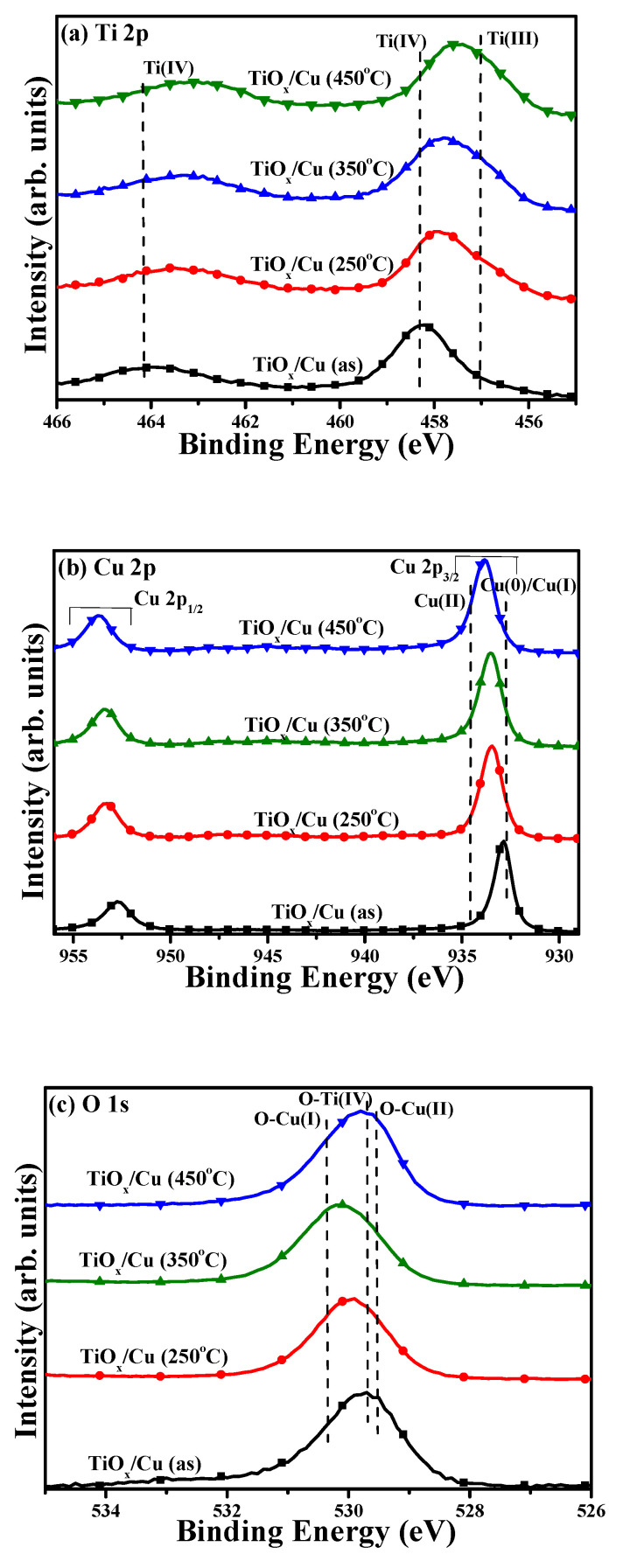
Binding energy spectra of the (**a**) Ti 2p, (**b**) Cu 2p, and (**c**) O 1s core levels measured from the surface of the as-deposited TiO_x_/Cu structure and the TiO_x_/Cu structures annealed at temperatures of 250 °C, 350 °C, 450 °C, respectively.

With the aim of investigating the annealing process on the changes of the chemical bond configurations, the compositions for the Ti, Cu, and O elements at the contact interface of these TiO_x_/Cu structures were examined using XPS measurements through Ar^+^ sputtering etching. Figure 7a–c respectively show the spectra of the binding energies related to the Ti 2p, Cu 2p, and O 1s core levels at the interface of the TiO_x_/Cu structure after etching by the Ar^+^ sputtering for 60 s. The intensity of the binding energy scales was normalized according to the maximum value in the corresponding XPS spectrum. The Ti 2p_3/2_ and Ti 2p_1/2_ signal at binding energies of 458.2 and 464.0 eV, respectively, with a difference of 5.8 eV, were measured from the interface of the as-deposited TiO_x_/Cu contact system (Figure 7a). The binding energies and their difference are consistence with the reports on the TiO_2_ film with anatase structure, which could be denoted as the bond configuration of Ti(IV) in the spectrum [1,30,31,32,33,34,35]. When the contact system annealed at a temperature of 250 °C, the Ti 2p_3/2_ signal shifted slightly to a lower binding energy of 458.0 eV, with a narrow difference between the signal of the Ti 2p_3/2_ and Ti 2p_1/2_ (~5.5 eV). This signal shifted clearly to 457.4 eV as the contact system annealed at 450 °C. As quoted from the bond configurations of the Ti species, the shift of the binding energy was ascribed to the appearance of the Ti(III) bond originating from the formation of the oxygen vacancies in the TiO_x_ film [5,36,37]. It also addresses how the higher the amount of oxygen vacancies that are distributed over the TiO_x_ film the lower the position of the binding energy of the Ti 2p_3/2_ signal. Based on the changes in the binding energy of the Ti 2p_3/2_ signal, it could be inferred that the oxygen vacancies distributed over the surface of the TiO_x_ film gradually increased as the annealing temperature on the structure increased. In the Cu 2p spectra (Figure 7b), two feature peaks emerged from the Cu 2p_3/2_ and Cu 2p_1/2_ spin-orbits, with almost the same difference in the binding energy (~19.8 eV), something which could be measured from all of the samples. The binding energy of the Cu 2p_3/2_ signal (~932.8 eV) obtained from the as-deposited TiO_x_/Cu structure was consistent with the signal measured from the metallic and/or low oxidized Cu states (denoted as Cu(0)/Cu(I) in the spectra) [38,39,40,41,42]. As the annealing temperature on this contact system increased, this signal gradually shifted toward a higher binding. The shift of the binding energy is related to the increase in the highly oxidized Cu state (denoted as Cu(II) at about 934.5 eV in this figure), which appeared at the interface of the TiO_x_/Cu structure [40,41,42,43,44]. This implies that the oxidization of the element Cu in the TiO_x_/Cu contact system after the annealing treatment can be traced with XPS analysis, though these Cu-oxidized states were little observed from their corresponding XRD patterns. In addition, the composition of these oxidized Cu states at the TiO_x_/Cu contact system could also be estimated by deconvoluting the Cu 2p_3/2_ signal into the overlapping peaks related to the Cu (II) and Cu(0)/Cu(I) states. According to the deconvolved area of the Cu(II) and Cu(0)/Cu(I) states, the composition of the highly oxidized state (Cu(II)) at the TiO_x_/Cu interface rose from 0.31 to 0.47 and then to 0.71 as the contact structure’s annealing temperature increased from 250 °C to 450 °C. The O 1s signal measured from the interface of the as-deposited TiO_x_/Cu structure (Figure 7c) at about 529.7 eV was correlated to the O-Ti(IV) chemical bond [5,32]. As the annealing treatment on the TiO_x_/Cu contact system, the O 1s signal became very significant (the original signal is about twice that of the as-deposited sample), revealing that a large number of the Cu atoms had been oxidized and come to dominate the spectra. The binding energies of the O 1s signal shifted from 529.9 eV to 530.1 eV as the annealing temperatures on the TiO_x_/Cu structure increased from 250 C to 350 °C. In reference to the reports on the oxidized states of the Cu element, the increase in the formation of the O-Cu(I) chemical bond (~530.4 eV) at the interface of the TiO_x_/Cu contact system was responsible for the shift of the binding energy [34,41,45]. It is worth noting that the O 1s signal clearly shifted to a low binding energy of about 529.8 eV as the contact system annealed at a temperature of 450 °C. This result implies that such an annealing treatment would cause the O-Cu(I) oxidized state to evolve into the highly oxidized state that was related to the O-Cu(II) chemical bond (denoted at a binding energy of 529.7 eV in the spectra) [38,45]. Incorporating the analysis on the Cu 2p and O 1s core levels, it can be seen that the Cu(0) state’s contact with the TiO_x_ film predominated over the interface of the as-deposited TiO_x_/Cu structure, while this contact gradually evolved into a TiO_x_/O-Cu(I) feature as the heterojunction structure oxidation occurred at 250 °C. As the annealed temperature reached 350 °C, more O-Cu(I) states were in contact with the TiO_x_ film. Eventually, the O-Cu(II) state became the dominant bonding type at the interface of the 450 °C-annealed TiO_x_/Cu structure. Based on the investigations of the chemical bond configurations at the interface of these TiO_x_/Cu structures, it was confirmed that the nanocomposite TiO_x_/Cu_x_O heterojunction structure could be realized simply by the annealing treatment. The amount of the Cu element in a highly oxidized state increased with the annealing temperature, while that of the Ti element in the surface of the TiO_x_ film evolved from a Ti(IV) to a Ti(III) state, revealing that the annealing process not only resulted in the oxidation of the Cu atoms but also caused the outdiffusion of the oxygen atoms from the TiO_x_ film into the Cu layer. As quoted from the reports, the photo-induced EHP from the Ti(III) oxidized state has also been demonstrated to be separated more effectively than that from the Ti(IV) oxidized state to result in a better photocatalytic activity [5,17].

**Figure 8:**
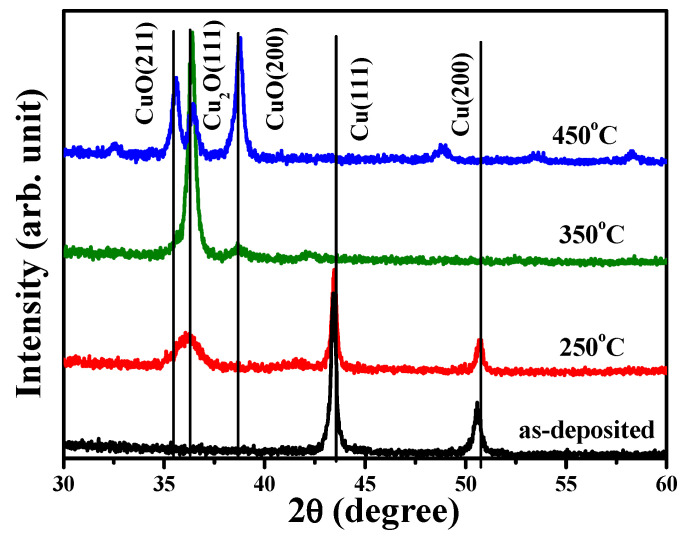
Crystalline evolutions of the as-deposited 200 nm Cu film and the films annealed at 250 °C, 350 °C, and 450 °C, conducted by XRD measurement (the correspondent crystal planes (hkl) are also given).

In order to further investigation the crystalline evolution of the single Cu layer affected by the annealing process, 200 nm thick Cu films were directly deposited onto the silicon substrate and processed at annealing temperatures of 250 °C, 350 °C, and 450 °C. Figure 8 depicts the crystalline evolutions of the as-deposited and annealed Cu films conducted by XRD measurement. The diffraction spectrum of the as-deposited Cu film exhibited only two dominant peaks at about 43.42° and 50.58°, respectively. These peaks could be identified as the face-centered cubic (fcc) structure of the crystal planes (hkl) of (111) and (200) (JCPDS No. 003-1018). As the Cu film annealed at a temperature of 250 °C under ambient oxygen, this thermal oxidation process resulted in another broad peak at about 36.06° other than the crystal planes correspondent with the metallic Cu structure in the XRD spectrum. According to the JCPDS No. 005-0667, this broad peak could be denoted as the oxidized structure of the cuprous oxide phase with the crystal plane (hkl) of (111). This transitionally oxidized Cu phase has also been reported to occur due to the insufficient activation energy between the copper and oxygen atoms [24,46,47]. The Cu_2_O(111) crystal plane became the dominant diffraction signal, while those of the crystal planes related to the metallic Cu (Cu(111) and Cu(200)) were absent in the XRD spectrum, as the single Cu film was annealed at 350 °C. In addition, a broad and weak diffraction peak at about 38.66°, identified as the crystal plane belonging to the cupric oxide crystalline phase (denoted as the crystal plane (hkl) of (200), per JCPDS No. 041-0254), also appeared in the XRD spectrum. As referred to in previous reports, the formation of this highly oxidized state of copper was correlated with the adequate thermal activation of copper and oxygen atoms [46,48]. This cupric oxide phase, with specific peaks correspondent to the crystal plane (hkl) of (211) and (200), then predominated over the XRD spectrum when the Cu film was annealed at 450 °C. According to the crystalline evolution of the thermally oxidized Cu layer and the chemical bonds of the oxidized Cu states at the interface of the annealed TiO_x_/Cu structures, it can be concluded that the metallic copper film was oxidized as the intermediate Cu_2_O phase while it was annealed at a low temperature and that the stable CuO phase formed as the metallic Cu layer annealed at an elevated temperature. The results are consistent with previous studies which demonstrate that the appearance of the phases for the oxidized Cu structure is closely related to the annealing temperature and ambient oxygen [22,48].

The electrical properties of the single Cu film oxidized at temperatures of 250 °C, 350 °C, and 450 °C are summarized in Table 2. The as-deposited Cu film possessed high electron carriers of about 2.49 × 10^22^ cm^−3^, with the mobility of 162 cm^2^/V s. A small decrease in electron carriers (~2.07 × 10^22^ cm^−3^) was measured as the Cu film oxidized at a temperature of 250 °C, which could be attributed to the intermediate Cu_2_O phase that was formed in the metallic Cu matrix. The conductive type, converted into *p*-type conduction with the hole carriers of about 6.03 × 10^16^ cm^−3^, was obtained as the Cu film was oxidized at an annealing temperature of 350 °C. This change in the conductive type could be connected to the metallic Cu being completely transformed into the oxidized Cu_2_O phase with the limited formation of the CuO phase, both of which have been demonstrated to be *p*-type semiconductors [23,24,47,49,50]. The structure that developed a composition of Cu_2_O and CuO phases as the Cu film oxidized at 450 °C also showed *p*-type conduction, with a similar hole concentration of about 3.66 × 10^16^ cm^−3^. The investigation on the crystalline structure of the oxidized Cu layer suggests that the TiO_x_/Cu contact system evolved into the TiO_x_/(Cu + Cu_2_O) contact system after being annealed at 250 °C under ambient oxygen. A completely oxidized structure constructed from the Cu_2_O phase with a little of the CuO phase was subsequently formed and contacted the TiO_x_ film when the annealed temperature on the structure reached 350 °C. Eventually, the oxidized CuO phase became the dominant structure contacted to the TiO_x_ film as the contact system annealed at a temperature of 450 °C. Although the conductivity of the anatase–TiO_x_ film prepared in this study was too low to be measured, it should demonstrate the *n*-type conductivity that is associated with the dominated oxygen-vacancy defects in the film [18,51,52]. Accordingly, it could be considered that the as-deposited and 250 °C-annealed TiO_x_/Cu contact systems formed a homogeneous heterojunction in *n*-type conduction. On the contrary, as *p*-type conductivity was measured from the single Cu film annealed at 350 and 450 °C, it can be suggested that the heterojunction structures with the opposite conductivity type (eq. *n*-TiO_x_/*p*-Cu_x_O) are formed as the TiO_x_/Cu contact system annealed at these two temperatures. Accordingly, an extra electrical field was built at the interfaces of the 350 °C- and 450 °C-annealed TiO_x_/Cu structures, which was beneficial for separating the EHPs generated at the contact interface under UV light irradiation. The products of the hydroxyl (·OH) and superoxide anion (·O_2_^−^) radicals originating from the electron and hole carriers, respectively, reacted with the ambient vapor and oxygen and thus could be enhanced due to the inhibition of the recombination of the photo-generated EHPs. As these ·OH and ·O_2_^−^ radicals form the primary functional group for catalyzing the decomposition of the organic compounds and for breaking the membrane of the bacteria on the TiO_x_ surface [53,54,55,56], the resulting 350 °C- and 450 °C-annealed TiO_x_/Cu contact systems that behaved as the *n/p* heterojunction structure therefore demonstrated superior activity in decomposing the MB solution and sterilizing the *E. coli*, as listed in Table 1 and Figure 6.

Except for the built-in voltage established at the interface of the 350 °C- and 450 °C-annealed TiO_x_/Cu structure for separating the photo-generated EHPs, the band offset between the anatase–TiO_x_ and the oxidized Cu states should also be noted, as it was crucial for driving the migration of these photo-generated carriers. The band offset between two materials is established from the discontinuity of the conduction (Δ*E_c_*) and valence (Δ*E_v_*) bands originating from the differences in the material’s energy bandgap (*E_g_*) and electron affinities (*qχ*). As quoted from [23,24,49,57], *p*-type Cu_2_O and CuO possess the energy bandgaps of about 2.2 eV and 1.7 eV, respectively, with electron affinities of about 3.3 eV and 3.8 eV. Incorporating the energy bandgap (~3.2 eV) and electron affinity (~3.9 eV) of the anatase–TiO_2_ phase, the conduction and valence band offsets between the TiO_2_ and Cu_2_O were 0.6 and 1.6 eV, respectively, while those of the band offsets between the TiO_2_ and CuO were 0.1 and 1.6 eV. Thus, the conduction band of the *p*-type Cu_2_O was more negative than that of the *n*-type TiO_2_, whereas the band offset of the conduction band between the *p*-type CuO and *n*-type TiO_2_ was relatively invisible. In contrast to the remarked difference in the band offset of the conduction band, the valence band of both the *p*-type Cu_2_O and CuO possessed almost the same anodic potential, which was less than that of the TiO_2_. This result suggests that the electron carriers at the *n*-TiO_x_/*p*-Cu_2_O interface in this study, generated under UV light irradiation, would be more prone to migrate to the TiO_x_ side than that of the carriers generated at the *n*-TiO_x_/*p*-CuO contact system, while a similar driving force appeared to cause the photo-generated hole carriers from the *n*-type TiO_x_ side to the *p*-type Cu_2_O or CuO interfaces. Thus, the recombination of the EHPs generated under UV light irradiation at the *n*-TiO_x_/*p*-Cu_2_O interface would be inhibited more effectively as a result of the *n*-TiO_x_/*p*-Cu_2_O structure possessing a superior band offset discontinuity than that of the *n*-TiO_x_/*p*-CuO structure.

**Figure 9:**
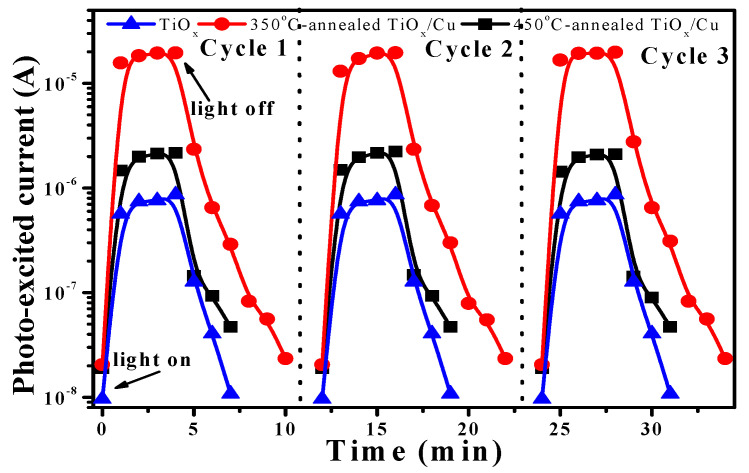
On–off current transient of the 350 °C- and 450 °C-annealed TiO_x_/Cu structures as well as the single TiO_x_ film irradiated by the UV lamp.

Figure 9 shows three cycles for the response of the photo-excited current as functions of the transient of the 350 °C- and 450 °C-annealed TiO_x_/Cu structures as well as the on–off current transient measured from the single TiO_x_ film. The photo-induced currents of these samples under UV light irradiation are reproducible and reliable, as could be seen from the three cycles’ on–off current transient. The single TiO_x_ film and the 350 °C- and 450 °C-annealed TiO_x_/Cu structures possessed dark currents of about 9.64 × 10^−9^ A, 2.05 × 10^−8^ A and 1.90 × 10^−8^ A, respectively. As the TiO_x_ film was illuminated by the UV light, the photo-generated carriers increased the current to about 8.62 × 10^−7^ A. In contrast to the TiO_x_ film, a large photo-excited current of approximately 1.96 × 10^−5^ A was measured from the 350 °C-annealed TiO_x_/Cu sample after being irradiated by the UV light. As the TiO_x_/Cu structure was annealed at 450 °C, a photo-induced current of about 2.16 × 10^−6^ A was measured. Incorporating the changes in currents of these structures before and after the UV light illumination, the on–off current transient (i.e., the ratio of the photo-excited current to the dark current) of the 450 °C-annealed TiO_x_/Cu structure (~113) was somewhat higher than that of the TiO_x_ film (~89), whereas an apparent increase could be obtained from the on–off current transient of the 350 °C-annealed TiO_x_/Cu structure (~953). Based on the above-mentioned investigation, the reason for the increase in the on–off current transient for those two annealed TiO_x_/Cu structures was the formation of the *n/p* heterojunction, which was helpful for the separation of the photo-generated carriers. Moreover, the way in which the 350 °C-annealed TiO_x_/Cu structure possesses a dominant *n*-TiO_x_/*p*-Cu_2_O interface with significant band offsets between the conduction and valence bands’ discontinuity could be a critical reason for the corresponding order of magnification that we find on the photo-induced current when compared with the 450 °C-annealed sample. This sample also demonstrated a longer transient time, as determined from the highest photo-excited current to the dark current (7 min), when compared with that of the 450 °C-annealed sample (4 min). Accordingly, the *n*-TiO_x_/*p*-Cu_2_O heterojunction predominated over the 350 °C-annealed TiO_x_/Cu structure, which significantly enhanced the photo-excited carriers and resulted in a longer carrier lifetime, exhibiting the quality behavior required to decompose the MB solution and inhibit *E. coli* growth under UV light irradiation. Based on the mechanism established from this study, the *p*-type conduction abundant in the Cu(I) oxidized state (ie. Cu_2_O crystalline structure) is essential for improving the photocatalytic activity of the TiO_x_/Cu contact system.

## 4. Conclusions

In this work, an oxidized TiO_x_/Cu structure was prepared simply by a thermal annealing process under ambient oxygen. Compared with the optical spectra of a single TiO_x_ film, both the as-deposited and annealed TiO_x_/Cu structures showed a reduction of about 20% for the optical transmittance within the visible wavelengths and a redshift of the absorption edge at the early UV wavelength. In addition, no compound related to the Ti-Cu alloys and also no trace of the Cu doping into the TiO_x_ matrix could be found from these TiO_x_/Cu contact systems. Although the optics and crystallinity of these annealed TiO_x_/Cu structures were almost similar to those of the as-deposited structure, their photocatalytic activities for decomposing the MB solution and inhibiting the growth of the *E. coli*. bacteria showed variation with the annealed temperatures. With the help of the studies on the chemical bond configurations at the interface of the TiO_x_/Cu structures as well the crystalline and electrical evolution of the single Cu layer under the same annealing temperatures, the photocatalytic activity of these TiO_x_/Cu contact systems was found to be deeply correlated to the changes of the oxidation states and conduction types of the upper-layered Cu. The type-conversion of the oxidized Cu layer at the annealed temperatures of 350 °C and 450 °C realized the formation of the nanocomposite *n*-TiO_x_/*p*-Cu_x_O heterojunction structures. Such a hetero-contact system was beneficial for the separation of the photogenerated EHPs over the TiO_x_ surface. Moreover, since the band offset between the *n*-TiO_x_/*p*-Cu_2_O structure was more significant than that of the *n*-TiO_x_/*p*-Cu_2_O structure, the *p*-type Cu_2_O crystalline phase that predominated over the 350 °C-annealed TiO_x_/Cu system results in the better separation of the photo-generated EHPs than that of the 450 °C-annealed sample, at which temperature most of the Cu_2_O structures had evolved into the CuO structures. The photocatalytic activity of the TiO_x_ film could further be optimized as the surface modified by a thin Cu layer and then oxidized at a temperature of 350 °C. The rate constant of the 350 °C-annealed TiO_x_/Cu structure derived from it decompose the MB solution under UV light irradiation was 38% higher than that of the single TiO_x_ film. Furthermore, this structure irradiated by the UV lamp for 1 h almost completely inhibited the growth of the *E. coli* bacteria for 24 h (the reduction percentage was about ~91%).

## Figures and Tables

**Table 1:** Rate constant (k) and reduction percentage (R) of the TiO_x_ film, and the as-deposited and annealed TiO_x_/Cu structures to decompose the MB solution and sterilize the *E. coli* under UV light irradiation.

	Cu	TiO_x_	As-Deposited	250 °C	350 °C	450 °C
*k* (min^−1^)	0.0038	0.0120	0.0091	0.0087	0.0165	0.0118
*R* (%)	37	64	54	43	91	68

*k* value of MB solution is 0.0036 min^−1^.

**Table 2:** Electrical properties as a function of the 200 nm Cu film annealed at various temperatures under ambient oxygen for 1 min.

	Conc. (cm^−3^)	μ_n_ (cm^2^/V s)	ρ (Ω cm)
As-deposited	−2.49 × 10^22^	1.62 × 10^2^	1.55 × 10^−6^
250 °C	−2.07 × 10^22^	1.54 × 10^2^	1.96 × 10^−6^
350 °C	6.03 × 10^16^	1.56	6.63 × 10^1^
450 °C	3.66 × 10^16^	2.67	6.39 × 10^1^

## Data Availability

The original contributions presented in this study are included in the article. Further inquiries can be directed to the corresponding author.
